# Churg-Strauss Syndrome in the Pediatric Age Group

**DOI:** 10.1097/WOX.0b013e3181626fde

**Published:** 2008-02-15

**Authors:** Yehia El-Gamal

**Affiliations:** 1 The Pediatric Allergy and Immunology Unit, Ain Shams University, Cairo, Egypt. 98 Mohamed Farid St, Cairo 11111, Egypt

**Keywords:** Churg-Strauss syndrome, ANCA, vasculitis, children

## Abstract

The rate of reporting of childhood Churg-Strauss syndrome (CSS) has increased lately because of either increased awareness to the disease or a real increase in incidence. It is defined as one of the antineutrophil cytoplasmic antibody-associated vasculi-tides, but the antineutrophil cytoplasmic antibody positivity is less reported in pediatric cases. The cause of CSS remains unknown. Several lines of evidence suggest genetic predisposition, which may entail inherited tendency to dysregulation of the cellular immune system. With the addition of leukotriene receptor antagonists to the treatment regimen of asthma, an association to CSS was presumed. However, the nature of this relationship remains to be elucidated. In addition, some environmental factors seem to provoke transient effects that resemble the disease. Patients' symptoms are defined by various degrees of eosinophilic inflammation and necrotizing vasculitis, which may affect any organ. Three clinical stages have been described in the clinical evolution of CSS: prodromal phase involving allergic rhinitis and asthma (usually without family history of atopy), a second phase that involves peripheral eosinophilia and eosinophilic tissue infiltration, and the hallmark of the final phase is systemic vasculitis. Pulmonary disease is a central feature of pediatric CSS, but other manifestations include skin lesions, testicular pain, hypertension, seizures, and nephropathy. More subtle presentations in children include cervical lymphadenopathy, acute abdominal pain, deep venous thrombosis, oral ulceration, multiple colonic ulcers, chorea, bilateral optic neuropathy, and retinal artery occlusions. Churg-Strauss syndrome patients usually respond well to corticoster-oid therapy. Several trials reported additional benefit from cyclopho-sphamide, azathioprine, and methotrexate, whereas the therapeutic effects of etanercept, plasma exchange, and intravenous immunoglo-bulin therapy are controversial. The relapse rate is approximately 25% to 30%, but corticosteroids have significantly increased survival, which now approaches greater than 75% at 5 years. However, there is limited information about survival or long-term outcome in childhood.

## Definition

This disease was first described in 1951 by Jacob Churg and Lotte Strauss as a syndrome consisting of asthma, eosinophilia, fever, and vasculitis of various organ systems. Their report was based on autopsy data and described diffuse vasculitis and extravascular granulomas with eosinophilic cores [1]. Churg-Strauss syndrome (CSS) is now defined as one of the antineutrophil cytoplasmic antibody (ANCA)-associated vasculitides. The predilection of disease manifestations for the respiratory tract, preferred affliction of small vessels including capillaries, and the frequent occurrence of ANCAs justify this grouping together with Wegener granulomatosis and microscopic polyangiitis. However, the allergic background in which the vasculitis presents, typically characterized by asthma and prominent peripheral blood and tissue eosinophilia, renders it unique among the primary systemic vasculitis syndromes [2].

## Epidemiology

The incidence of CSS in adults is estimated at 2.4 per million per year [3]. Because of its rarity, the incidence of the disease in the pediatric age group is unknown. It is conceivable that patients may go undiagnosed because of the lack of specificity, low index of suspicion, and invasiveness involved with pathological confirmation [4]. Before 1999, only 10 cases of childhood CSS (4-16 years) had been reported [5]. The rate of reporting has increased lately because of either increased awareness to the disease or a real increase in incidence caused by hypersensitivity to drugs or other environmental agents. Not less than 15 pediatric cases have been cited in literature during the past 8 years [4,6-18]. The youngest reported patients with CSS were 2 years old [13,19].

Few data are available regarding racial variations in occurrence or severity of manifestations. It has been suggested that CSS shares with other systemic vasculitides the tendency toward greater prevalence in whites [20]. Literature reveals that a significant number of reports are arising from Japan, [9,12,15] suggesting a possible geographic or ethnic predominance [4]. In adults, males are slightly more likely than females to develop the syndrome [21]. This does not seem to apply to pediatric CSS.

## Pathogenesis

Churg-Strauss syndrome remains a rare disease with a poorly understood pathogenesis. No data have been reported regarding the role of immune complexes or cell-mediated mechanisms in this disease, although autoimmunity is evident by the presence of hypergammaglobulinemia, rheumatoid factor, and ANCA [21].

Antineutrophil cytoplasmic antibodies (ANCAs) with perinuclear staining pattern (p-ANCAs) are detected. Phenotypically, ANCA-positive and ANCA-negative CSS might differ. The association of ANCA positivity with clinical symptoms that indicate inflammation and necrosis of small vessels might characterize a predominantly vasculitic pattern [22]. The ANCAs may promote polymorphonuclear cell adherence to vascular endothelial cells [20]. In vitro, ANCAs can further activate primed neutrophils to release reactive oxygen species and lytic enzymes, and in conjunction with neutro-phils, can damage and lyse endothelial cells [23]. An independent or adjuvant role in this activation may be played by tumor necrosis factor (TNF) [20].

It was shown that stimulated peripheral blood mono-nuclear cells from patients with CSS secrete significantly increased amounts of interleukin 5 (IL-5) compared with healthy controls, suggesting that IL-5 contributes substantially to the development of eosinophilia in CSS. Variations in the balance between T_H_1 and T_H_2 cytokines at different disease stages could contribute to the distinct clinical courses seen in patients with CSS, which can range from prominent T_H_1-mediated generalized vasculitis and granulomatous inflammation on 1 end of the spectrum to T_H_2-mediated systemic hypereosinophilia on the other [24]. Occasionally, CD20[+ ]B lymphocytes are found in the inflammatory exudate, and deposits of immunoglobulin G (IgG), IgE, and C3d may be detected [25]. Cytokines undoubtedly participate in this auto-immune process. Patients with CSS have markedly increased serum levels of interferon alfa and IL-2 and moderate increases of TNF-α and IL-1β similar to those observed in polyarteritis nodosa. Elevations of serum IL-6 concentrations have been shown to precede the rise in serum rheumatoid factors that may accompany the onset of an exacerbation of Churg-Strauss vasculitis in adults [20]. Soluble CD95, which was identified as a survival factor for eosinophils rescuing eosinophils from apoptosis in vitro, was found overexpressed in CSS, which may suggest that it may be mechanistically involved in the disease [26].

## Pathology

Patients' symptoms are defined by various degrees of eosinophilic inflammation and necrotizing vasculitis, which may affect any organ [27]. The vasculopathy of CSS is predominantly an arteriopathy tending to affect small- and medium-sized arteries much more than arterioles, veins, or capillaries. This predilection also is found in polyarteritis nodosa and some other conditions. However, the predominance of eosinophils sets CSS apart from these other conditions. Epithelioid and giant cells also are found in the inflammatory exudate of patients with CSS. The inflammatory arteriopathy evolves into granulomatous fibrinoid necrosis of the vascular media. The granulomatous material surrounds altered vascular elastic fibers, collagen, and acellular pigmented debris, which is helpful in pathologically distinguishing 1 form of granulomatous vasculitis from another. Churg-Strauss syndrome is associated with red collagenolytic granulomas [20].

The number of reported childhood cases remains too small for adequate comparisons between the histological features of adult and childhood disease [4]. Specific pulmonary histological findings in pediatric CSS have been described only once, briefly, in a report describing granulomas and angiitis with focal necrosis and eosinophilic infiltration in a 14-year-old adolescent boy [28]. Lung biopsies from 2 patients showed aggregates of intra-alveolar eosinophils, "eosinophilic abscesses" constituting eosinophilic pneumonia. The biopsy specimen of one of them revealed extravascular microgranu-lomas filled with eosinophils and their breakdown products [4]. These changes were described in the original report of CSS by Churg and Strauss, [1] and although not consistently found in adult CSS, they are thought to be specific for the disease.

## Etiology

The cause of CSS remains unknown. Several lines of evidence suggest genetic predisposition, which may entail inherited tendency to dysregulation of the cellular immune system [20]. Recent studies have demonstrated primary genetic causes for hypereosinophilia [29]. There is significant clinical overlap between the hypereosinophilic syndromes and CSS. It is possible that primary genetic disorders may tend especially to underlie disease with early (childhood) presentations [4]. Myeloperoxidase (MPO)-directed ANCAs that are found in patients with Churg-Strauss disease are also found in microscopic polyangiitis. It was suggested that a mutation in exon 11 of the CD18 gene may be permissive for the elaboration of anti-MPO antibodies in either condition [20].

With the recent addition of leukotriene receptor antagonists (LRAs) to the treatment regimen of asthma, an association to CSS was presumed. The association of CSS with LRAs therapy is found in perhaps half of the cases, and the addition of those drugs was made within 3 months before the CSS manifestations in three quarters of these patients [30]. However, the nature of this relationship remains to be elucidated. The occurrence of CSS in asthmatic patients receiving leukotriene modifiers seems to be related to unmasking of an underlying vasculitic syndrome that is initially clinically recognized as moderate to severe asthma and treated with corticosteroids [31-33]. However, leukotriene modifier-associated Churg-Strauss syndrome was reported in steroid-naive asthmatics [34] and in a patient with no recent use of oral steroids [35]. In addition, a patient with CSS who experienced a clinical relapse after treatment with montelukast was reported by Solans et al [36]. These data may suggest an allergic response to the drug.

Almost 150 cases of LRA-associated CSS have been reported by the US Food and Drug Administration until 2001 [37]. In addition, a summary of 24 cases suggested an association of CSS with LRA [38]. In a recently reported series of cases, however, a history of exposure to a LRA preceded the CSS-related symptoms in only 13 of 23 patients, and the time of disease progression from asthma to CSS was not affected by the exposure [39]. The youngest reported patient with CSS after a prolonged course of montelukast was 7 years old [14].

Conen et al [40] reported a case of CSS in an asthmatic patient who developed severe obstructive symptoms and progressive heart failure after 2 sequential exposures to montelukast. The time course of events strongly suggested a direct etiologic role for the drug. They tried to explain the mechanism by which LRAs might have caused the eosinophilic vasculitis, but it remained unclear. Hypersensitivity reactions tend to cause a leukocytoclastic rather than a granulomatous vasculitis. Therefore, a hypersensitivity reaction to LRAs seems unlikely. Leukotriene B4 (LTB4), a powerful chemoattractant of eosinophils, is not inhibited by LRAs [41]. This could lead to increased plasma levels of LTB4 and trigger eosinophilic inflammation. However, CSS has also been reported in association with inhibitors of 5-lipoxygenase (eg, zileuton), which also block LTB4 [31].

On the other hand, 7 cases of complete and incomplete forms of CSS not related to LRAs were reported by Bili et al, [42] who concluded that CSS can happen even in patients with mild asthma using only inhaled corticosteroids. Although safe in most patients, asthmatic patients who are prescribed LRAs should be monitored carefully [40,43]. The previously described steroid-sparing effect of LRAs is controversial because a randomized controlled trial revealed that corticosteroids cannot be reduced in patients with persistent moderate to severe asthma despite administration of LRAs [44].

Carbamazepine [45] and macrolide antibiotics [46] are other drugs that have been implicated as provocative causes of CSS. In addition, some environmental factors seem to provoke transient effects that resemble the disease but do not represent a chronic and self-perpetuating disease. The inhalation of fungal spores, such as those produced by *Actinomyces *and *Aspergillus *species, has been implicated in the pathogenesis of some cases. Exposure to pigeons and the molds associated with their roosts may provoke the development of CSS [20]. Smoking of free-base cocaine was documented as a circumstance preceding individual bouts of an illness identical to CSS [47].

## Clinical picture

Three stages have been described in the clinical evolution of CSS. The typical prodromal phase involves allergic rhinitis and asthma (usually without family history of atopy) that can be protracted for many years. The second includes peripheral eosinophilia and eosinophilic tissue infiltration. The hallmark of the final phase is systemic vasculitis, which if left untreated, can be fatal. It may involve the skin, lungs, musculoskeletal structures, gastrointestinal tract, kidneys, and central and peripheral nervous systems [4,48]. The first phase usually lasts for 3 to 10 years before the development of systemic vasculitis [7]. In other words, asthma is just the presenting manifestation of CSS that is actually a systemic vasculitic disorder.

Pulmonary disease is a central feature of CSS, and the reported range of pulmonary manifestations in childhood is widening. It may take the form of transitory patchy or diffuse pulmonary infiltrates, nodular infiltrates, or pleural effusion, often with high fever and severe systemic manifestations [4,5,7-9,11,15,48-51]. Eosinophilic pleural effusion was the first clue to the diagnosis of CSS in a 16-year-old adolescent girl [4]. Eosinophils in a pleural effusion are nonspecific and can be found in various conditions including trauma, pneumothorax, malignancy, congestive heart failure, infections, and rheumatologic conditions [52,53]. Although rare, CSS should be considered in the differential diagnosis of an eosinophilic effusion [54]. Less common pulmonary manifestations include hemoptysis, [7] chest pain, and pulmonary infarction [9].

Cardiovascular disease occurs in approximately half of the patients with CSS, irrespective of age [4]. Specific cardiac involvement in CSS includes pericarditis and, less commonly, tamponade and myocarditis [9]. Congestive heart failure/myocardial infarction accounted for 48% of all deaths in 1 series [48]. Severe cardiomyopathy, [51] occasionally with peri-cardial effusion, [7] has been reported in children and was fatal in a case [19]. All patients with CSS should be screened for signs of cardiac involvement [4].

Skin manifestations are a common early presentation of CSS in childhood. They range from discrete to diffuse patchy, petechial, or purpuric lesions and can be itchy, edematous, or tender [9,13,15,51]. A 12-year-old child with CSS experienced typical cutaneous manifestations including palpable purpura, skin nodules, urticarial rash, and rare lesions, such as finger tip vesicles, infiltrated papules, and aseptic pustules [7].

The most common neurological manifestation of CSS is mononeuritis multiplex. Initial mononeuritic findings often progress to asymmetrical polyneuropathy, which is restricted to the limbs. Both motor and sensory deficits are detectable, especially in the legs. Sensory disturbances may include hypoesthesia or hyperesthesia, allodynia, and pain [20,30]. Cranial nerve palsies develop infrequently [7,48,50,55]. Vasculitis of muscle develops in slightly more than 20% of patients. Childhood-onset CSS is less likely to be complicated by central nervous system (CNS) vasculitis than cases that develop in middle-aged individuals [20]. One girl with CSS, who was in the early years of the second decade of her life, developed chorea [56].

Some nonrespiratory symptoms, such as paresthesia and acalculous cholecystitis, in a patient with asthma should alert the physician to consider the possibility of CSS [57]. Renal involvement in CSS is usually benign and rarely progresses to renal failure. Two documented cases of renal disease and 2 with proteinuria were encountered among 10 reported pediatric patients, [5] but this was less frequent in the more recent reports.

In addition to clinical features seen in adults, reported symptoms in children included testicular pain, hypertension, and seizures [58]. More subtle presentations of CSS in children include discrete cervical lymphadenopathy, [6] acute abdominal pain, [8] deep venous thrombosis, [9] multiple colonic ulcers, [10] sudden painless loss of vision with bilateral optic neuropathy and retinal artery occlusions, [17] and oral ulceration [59]. Some rare instances of limited CSS that involved allergic granulo-matosis and angiitis in the absence of asthma and blood eosinophilia were reported [60,61].

## Diagnosis

The American College of Rheumatology developed criteria for the diagnosis of CSS in 1990. The presence of 4 or more of these 6 criteria yielded a sensitivity of 85% and a specificity of 99.7%[62]:

1. Asthma: history of wheezing or diffuse high-pitched expiratory rhonchi.

2. Eosinophilia: greater than 10% on differential white blood cell count.

3. Mononeuropathy or polyneuropathy: development of mononeuropathy, multiple mononeuropathies, or poly-neuropathy (glove/stocking distribution) attributable to systemic vasculitis.

4. Pulmonary infiltrates that are nonfixed: migratory or transitory pulmonary infiltrates attributable to vasculitis.

5. Paranasal sinus abnormality: history of acute or chronic paranasal sinus pain or tenderness or radiographic opacification of the paranasal sinuses.

6. Extravascular eosinophils: biopsy including artery, arteriole, or venule showing accumulations of eosino-phils in extravascular areas.

A classification tree was also constructed with 3 selected criteria: asthma, eosinophilia greater than 10% on differential white blood cell count, and history of documented allergy other than asthma or drug sensitivity. For the tree classification, the sensitivity was 95%, and the specificity was 99.2% [62].

The 1994 Chapel Hill Consensus definitions aimed to reach a consensus on the names of the major noninfectious vasculitides and provided working definitions of 10 different types of vasculitis based principally on the size of the affected vessel. Churg-Strauss syndrome is classified with Wegener granulomatosis under small-vessel necrotizing vasculitis [63].

From a series of 16 patients and a review of literature on 138 patients, Lanham et al [48] proposed diagnostic criteria for CSS involving asthma, eosinophilia in excess of 1.5 *3 *10^9^/L, and systemic vasculitis involving 2 or more extrapulmonary sites. Mean values for absolute eosinophil counts are in the range of 5000 to 9000/mL, but in rare instances, counts may exceed 100,000/mL. Treatment of asthmatic manifestations of the first stage of CSS with corticosteroids may obscure this characteristic finding. The prompt resolution of eosinophilia with corticosteroid treatment is itself quite characteristic of CSS, and the intermittent elevations of eosinophil counts during the third phase of the disease may presage a relapse of systemic vasculitis [20].

A high index of suspicion is a key to the diagnosis of CSS in childhood. Specifically, evidence of neuropathy or cardio-myopathy in conjunction with a previous history of asthma and sinusitis should alert the practitioner to this diagnosis even in children and young adults [4]. Of the diagnostic laboratory criteria, only ANCA seems to be significantly different between children and adults. Nearly half of the adults with CSS are positive for ANCA [50]. In contrast, neither of 2 patients described in 1 report [4] was positive for ANCA, nor did 9 of 10 children in a pediatric series [3].

The concentration of ANCA may be used to monitor disease activity and assist decision making related to therapeutic dose reduction. The addition of monitoring levels of antibodies to specific neutrophil enzymes--such as elastase, proteinase 3, or MPO--may enhance the reliability of ANCA changes in predicting changes in disease activity. Anti-MPO antibodies are characteristic in CSS (associated with p-ANCA), whereas anti-proteinase 3 (PR3) are more specific for Wegener granulomatosis (associated with c-ANCA) [64]. Animal model studies provided some evidence of a direct pathogenic effect of passively transferred MPO and proteinase 3-specific ANCAs in inducing pauci-immune glomerulonephritis and vasculitis [65,66].

In its active state, CSS may be associated with markedly increased levels of soluble IL-2 receptor, eosinophil cationic protein, and soluble thrombomodulin, indicating T-cell and eosinophil activation [67]. Serum IgE concentrations are elevated in three quarters of patients in the second or third phase. Erythrocyte sedimentation rate and other indices suggestive of the presence of acute-phase reactants may be also elevated [20].

Chest radiographs demonstrate pulmonary infiltrates in at least half of the patients in the second phase of Churg-Strauss disease and a greater percentage in the third phase of the disease. The areas of consolidation can be symmetric or asymmetric and may have a peripheral distribution similar to that seen in chronic eosinophilic pneumonia. Less common manifestations include small or large nodules, unilateral or bilateral pleural effusions, and hilar lymphadeno-pathy [48,68,69]. Lungs may be hyperinflated. Nonsegmental reticulonodular opacities without cavitation may be found. Bronchial walls may be thickened, and enlarged intrapulmon-ary lymph nodes may be found in some cases [20].

High-resolution computed tomography frequently demonstrates bilateral ground-glass opacities with or without associated areas of consolidation [70]. Increased vascular wall caliber also may be discerned as well as enlarged hilar or mediastinal lymph nodes representing an opportunity for diagnostic biopsy. Imaging of the heart may reveal cardiome-galy or pericardial effusion. Magnetic resonance imaging (MRI) of the brain in patients with CNS manifestations may reveal vascular territory infarction, with or without hemorrhage [20]. Diffusion-weighted echoplanar MRI is said to detect small and active ischemic changes not visible on conventional MRI and may clearly discriminate cytotoxic from vasogenic edema in patients with cerebral vasculitis or vasculopathy [71].

Electrophysiological studies of peripheral nerves may reveal deficits referable to both myelinated and unmyelinated sensory and motor fibers, especially those subserving the lower extremities. The absence of conduction blocks may be helpful in distinguishing Churg-Strauss mononeuritis multiplex from chronic inflammatory demyelinating polyneuropathy [20]. Glomerulonephritis is not as common or severe as in Wegener granulomatosis, but when present, it is usually focal and segmental and indistinguishable from other forms of the so-called pauci-immune (without significant tissue deposition of immune complexes) glomerulonephritis [21].

The seriousness of this disease and its impact on longevity require tissue diagnosis and should not rely solely on the American College of Rheumatology criteria. The latter could potentially lead to overdiagnosis of borderline cases. Transbronchial biopsies in young patients yield a minute amount of tissue; therefore, it was suggested that such samples be used to confirm a diagnosis when appropriate but should not be used to rule the diagnosis out before a larger tissue sample can be analyzed [4].

## Differential Diagnosis

There are some similarities between CSS and each of the following [20,21]:

• Asthma

• Eosinophilic pneumonia

• Goodpasture syndrome

• Hypereosinophilic syndrome

• Microscopic polyangiitis

• Polyarteritis nodosa

• Wegener granulomatosis

• Allergic bronchopulmonary aspergillosis

• Strongyloidosis with hyperinfection syndrome

• Loeffler syndrome

• Tropical pulmonary eosinophilia

• Cryoglobulinemia

• Acute mesenteric ischemia

• Crescentic glomerulonephritis

## Treatment

Churg-Strauss syndrome patients usually respond well to corticosteroid therapy often started in doses of 1 mg/kg per day. The acute exacerbations usually require treatment with high doses of steroids for several weeks [7,48]. Intravenous administration of methylprednisolone at doses of 15 mg/kg on 1 to 3 successive mornings is one of the most common initial approaches to severe cases [21].

Several trials reported benefit from cyclophosphamide, azathioprine, and methotrexate in controlling disease progression or reducing relapse [72,73]. Pulse intravenous cyclopho-sphamide therapy in combination with corticosteroids seems to diminish the risk for various adverse effects seen in patients receiving oral cyclophosphamide daily [20].

Plasma exchange has been tried but has not added benefit to the treatment of patients who were treated with prednisone or cyclophosphamide. This is based on a meta-analysis of 140 patients with glomerulonephritis in CSS and microscopic polyangiitis [74].

Intravenous immunoglobulin was considered to be of some value in Churg-Strauss vasculitis, but a randomized placebo-controlled trial of 34 patients with ANCA-positive vasculitis suggested only a transient improvement [75]. A recent review of evidence by members of the Primary Immunode-ficiency Committee of the American Academy of Allergy, Asthma and Immunology revealed that using intravenous immunoglobulin in ANCA-positive vasculitis might provide benefit with an evidence category of III and a low recommendation strength (D). Such lack of convincing evidence prevents a recommendation for its routine use [76].

There is compelling evidence that TNF-α plays an important role in the pathogenesis of ANCA-associated vasculitis. Clinical trials that used TNF-α blockers in patients with ANCA-associated vasculitis gave mixed results. Importantly, in a large-scale randomized trial, treatment with etanercept was found ineffective and resulted in an excess of treatment-related morbidity [77,78].

Several case reports have described infliximab and rituximab use in patients with steroid-dependent CSS [79-81]. Interferon alfa is said to exert a beneficial effect by producing a dose-dependent decrease in the blood eosino-phil count [82]. Other medications tried include thalidomide [20] in male patients, mycophenolate mofetil, [83] and anti-IgE (omalizumab) therapy [84].

## Prognosis

A 5-factor score determining the poor prognosis in CSS was described by Guillevin et al [72]. It included renal failure, severe proteinuria, severe gastrointestinal involvement, cardiomyo-pathy, and CNS involvement. Short duration from the onset of asthma to the onset of systemic vasculitis is probably another poor prognostic factor [7]. In general, as the number of systems involved increases, a higher mortality is observed [4]. In addition, leukopenia caused by immunosuppressive therapy enhances risk for sepsis and hence morbidity and mortality [85].

Patients demonstrating a favorable response usually retain an independent existence on steroid maintenance therapy. The relapse rate is approximately 25% to 30% [20]. Corticosteroids have significantly increased survival, which now approaches greater than 75% at 5 years [3]. The longest reported survival is 37 years [86]. Obviously, there is limited information about survival or long-term outcome in childhood.

## References

[B1] Churg J Strauss L Allergic granulomatosis, allergic angiitis, and periarteritis nodosa Am J Pathol 1951 1 277 301 14819261 PMC1937314

[B2] Keogh KA Specks U Churg-Strauss syndrome: update on clinical, laboratory and therapeutic aspects Sarcoidosis Vasc Diffuse Lung Dis 2006 1 3 12 16933465

[B3] Cottin V Cordier JF Churg-Strauss syndrome Allergy 1999 1 5 10435467 10.1034/j.1398-9995.1999.t01-1-00091.x

[B4] Boyer D Vargas SO Slattery D Rivera-Sanchez YM Colin AA Churg-Strauss syndrome in children: a clinical and pathologic review Pediatrics 2006 1 5 10.1542/peds.2006-0113 16894009

[B5] Louthrenoo W Norasetthada A Khunamornpong S Sreshthaputra A Sukitawut W Childhood. Churg-Strauss syndrome J Rheumatol 1999 1 5 10381061

[B6] Casey M Radel E Ratech H Lymph node manifestations of limited Churg-Strauss syndrome J Pediatr Hematol Oncol 2000 1 5 10.1097/00043426-200009000-00018 11037864

[B7] Wang SJ Yang YH Lin YT Tsai MJ Chiang BL Childhood Churg-Strauss syndrome: report of a case J Microbiol Immunol Infect 2000 1 5 11269373

[B8] Berlioz M Triolo V Sirvent N Albertini M Churg-Strauss syndrome revealed by acute abdominal pain Pediatr Pulmonol 2001 1 5 10.1002/ppul.1950322304 11416881

[B9] Ikemoto Y Kohdera U Uraoka M Teraguchi M Okamura A Kobayashi Y Pulmonary infarction and deep venous thrombosis in a 13-year-old boy with Churg-Strauss syndrome Pediatr Int 2001 1 5 10.1046/j.1442-200x.2001.01400.x 11472598

[B10] Lin TL Wang CR Liu MF Multiple colonic ulcers caused by Churg-Strauss syndrome in a 15-year-old girl Clin Rheumatol 2001 1 5 10.1007/pl00011209 11642519

[B11] Richeldi L Rossi G Ruggieri MP Corbetta L Fabbri LM Churg-Strauss syndrome in a case of asthma Allergy 2002 1 5 12100309 10.1034/j.1398-9995.2002.23618.x

[B12] Kobayashi S Ishizuka S Tamura N Takaya M Kaneda K Hashimoto H Churg-Strauss syndrome (CSS) in a patient receiving pranlukast Clin Rheumatol 2003 1 5 10.1007/s10067-003-0791-5 14677037

[B13] Tomac N Yuksek M Kunak B Ertan U Igde M Churg-Strauss syndrome: a patient report in infancy Clin Pediatr (Phila) 2003 1 5 10.1177/000992280304200412 12800734

[B14] Turvey SE Vargas SO Phipatanakul W Churg-Strauss syndrome in a 7-year-old receiving montelukast and inhaled corticosteroids Ann Allergy Asthma Immunol 2003 1 5 10.1016/S1081-1206(10)62156-4 12602680

[B15] Fujita K Yamato K Kurihara T A case of allergic granulomatosis and angiitis (Churg-Strauss syndrome) in a 15-year-old girl [in Japanese] Nihon Kokyuki Gakkai Zasshi 2004 1 5 15500154

[B16] Kashef S Alyasin S Kiani M Amin R Alborzi A Churg-strauss syndrome in an 8-year-old girl Iran J Allergy Asthma Immunol 2004 1 5 17301391

[B17] Partal A Moshfeghi DM Alcorn D Churg-Strauss syndrome in a child: retina and optic nerve findings Br J Ophthalmol 2004 1 5 10.1136/bjo.88.1.5 15205253 PMC1772240

[B18] Olowu WA Nephropathy, polyneuropathy, and gastroenteritis in a child with Churg-Strauss syndrome Clin Rheumatol 2007 1 5 10.1007/s10067-006-0288-0 16897116

[B19] Rabusin M Lepore L Costantinides F Bussani R A child with severe asthma Lancet 1998 1 5 10.1016/S0140-6736(05)78098-3 9433427

[B20] Rust RS Worrell TE Churg-Strauss disease eMedicine Specialties: Pediatric Neurology Available at: http://www.emedicine.com/neuro/topic501.htm. Accessed August 20, 2007

[B21] Farid-Moayer M Sessoms SL Churg-Strauss syndrome eMedicine Specialties: Rheumatology Available at: http://www.emedicine.com/med/topic2926.htm. Accessed August 20, 2007

[B22] Sable-Fourtassou R Cohen P Mahr A Antineutrophil cytoplasmic antibodies and the Churg-Strauss syndrome Ann Intern Med 2005 1 5 10.7326/0003-4819-143-9-200511010-00006 16263885

[B23] Kallenberg CG Heeringa P Stegeman CA Mechanisms of disease: pathogenesis and treatment of ANCA-associated vasculitides Nat Clin Pract Rheumatol 2006 1 5 10.1038/ncprheum0355 17133251

[B24] Hellmich B Csernok E Gross WL Proinflammatory cytokines and autoimmunity in Churg-Strauss syndrome Ann N Y Acad Sci 2005 1 5 10.1196/annals.1361.053 16126951

[B25] Hattori N Ichimura M Nagamatsu M Clinicopathological features of Churg-Strauss syndrome-associated neuropathy Brain 1999 1 pt 3 5 10094252 10.1093/brain/122.3.427

[B26] Muschen M Warskulat U Perniok A Involvement of soluble CD95 in Churg-Strauss syndrome Am J Pathol 1999 1 5 10.1016/S0002-9440(10)65191-7 PMC1866905 10487849

[B27] Keogh KA Specks U Churg-Strauss syndrome Semin Respir Crit Care Med 2006 1 5 10.1055/s-2006-933668 16612766

[B28] Treitman P Herskowitz JL Bass HN Churg-Strauss syndrome in a 14-year-old boy diagnosed by transbronchial lung biopsy Clin Pediatr (Phila) 1991 1 5 10.1177/000992289103000402 1914353

[B29] Tefferi A Modern diagnosis and treatment of primary eosinophilia Acta Haematol 2005 1 5 10.1159/000085558 15995325

[B30] Kawakami T Soma Y Kawasaki K Kawase A Mizoguchi M Initial cutaneous manifestations consistent with mononeuropathy multiplex in Churg-Strauss syndrome Arch Dermatol 2005 1 5 10.1001/archderm.141.7.873 16027304

[B31] Wechsler ME Finn D Gunawardena D Churg-Strauss syndrome in patients receiving montelukast as treatment for asthma Chest 2000 1 5 10.1378/chest.117.1.5 10712995

[B32] Kemp JP Recent advances in the management of asthma using leukotriene modifiers Am J Respir Med 2003 1 5 10.1007/BF03256645 14720013

[B33] Cuchacovich R Justiniano M Espinoza LR Churg-Strauss syndrome associated with leukotriene receptor antagonists (LTRA) Clin Rheumatol 2007 1 5 10.1007/s10067-006-0288-0 17256102

[B34] Green RL Vayonis AG Churg-Strauss syndrome after zafirlukast in two patients not receiving systemic steroid treatment Lancet 1999 1 5 10073523 10.1016/S0140-6736(99)00565-6

[B35] Villena V Hidalgo R Sotelo MT Martin-Escribano P Montelukast and Churg-Strauss syndrome Eur Respir J 2000 1 5 10.1183/09031936.00.15100500 10759464

[B36] Solans R Bosch JA Selva A Orriols R Vilardell M Montelukast and Churg-Strauss syndrome Thorax 2002 1 5 10.1136/thorax.57.1.5 11828052 PMC1746257

[B37] Donohue JF Montelukast and Churg-Strauss syndrome Chest 2001 1 5 10.1378/chest.119.1.5 11171764

[B38] Guilpain P Viallard JF Lagarde P Churg-Strauss syndrome in two patients receiving montelukast Rheumatology 2002 1 5 12011377 10.1093/rheumatology/41.5.535

[B39] Keogh AK Specks U Churg-Strauss syndrome: clinical presentation, antineutrophil cytoplasmic antibodies, and leukotriene receptor antagonists Am J Med 2003 1 5 10.1016/s0002-9343(03)00359-0 12967693

[B40] Conen D Leuppi J Bubendorf L Ronsdorf A Tamm M Hauser T Montelukast and Churg-Strauss syndrome Swiss Med Wkly 2004 1 5 10.4414/smw.2004.10661 15340881

[B41] Munoz NM Douglas I Mayer D Herrnreiter A Zhu X Leff AR Eosinophil chemotaxis inhibited by 5-lipoxygenase blockade and leukotriene receptor antagonism Am J Resp Crit Care Med 1997 1 5 10.1164/ajrccm.155.4.9105085 9105085

[B42] Bili A Condemi JJ Bottone SM Ryan CK Seven cases of complete and incomplete forms of Churg-Strauss syndrome not related to leukotriene receptor antagonists J Allergy Clin Immunol 1999 1 5 10.1016/S0091-6749(99)70106-5 10550753

[B43] Michael A Murphy D Montelukast-associated Churg-Strauss syndrome Age Ageing 2003 1 5 10.1093/ageing/afg076 12958007

[B44] Robinson DS Campbell D Barnes PJ Addition of leukotriene antagonists to therapy in chronic persistent asthma: a randomized double-blind placebo-controlled trial Lancet 2001 1 5 10.1016/S0140-6736(00)05113-8 11438132

[B45] Imai H Nakamoto Y Hirokawa M Akihama T Miura AB Carbamazepine-induced granulomatous necrotizing angiitis with acute renal failure Nephron 1989 1 5 10.1159/000185332 2918954

[B46] Dietz A Hubner C Andrassy K Laryngorhinootologie 1998 1 5 [in German]

[B47] Orriols R Munoz X Ferrer J Huget P Morell F Cocaine-induced Churg-Strauss vasculitis Eur Respir J 1996 1 5 10.1183/09031936.96.09010005 8834352

[B48] Lanham JG Elkon KB Pusey CD Hughes GR Systemic vasculitis with asthma and eosinophilia: a clinical approach to the Churg-Strauss syndrome Medicine (Baltimore) 1984 1 5 10.1097/00005792-198403000-00001 6366453

[B49] Lanham JG Churg-Strauss syndrome Br J Hosp Med 1992 1 5 1617339

[B50] Guillevin L Cohen P Gayraud M Lhote F Jarrousse B Casassus P Churg Stauss syndrome. Clinical study and long term follow up of 96 patients Medicine 1999 1 5 10.1097/00005792-199901000-00003 9990352

[B51] Reinhard H Lindinger A Geib-Konig R Graf N Klin Padiatr 1999 1 5 [in German] 10.1055/s-2008-1043826 10572904

[B52] Adelman M Albelda SM Gottlieb J Haponik EF Diagnostic utility of pleural fluid eosinophilia Am J Med 1984 1 5 10.1016/0002-9343(84)90428-5 6496547

[B53] Wysenbeek AJ Lahav M Aelion JA Kaufmann L Eosinophilic pleural effusion: a review of 36 cases Respiration 1985 1 5 10.1159/000194802 4023441

[B54] Erzurum SC Underwood GA Hamilos DL Waldron JA Pleural effusion in Churg-Strauss syndrome Chest 1989 1 5 10.1378/chest.95.6.1357 2721280

[B55] Sehgal M Swanson JW DeRemee RA Colby TV Neurologic manifestations of Churg-Strauss syndrome Mayo Clinic Proc 1995 1 5 10.4065/70.4.337 7898138

[B56] Kok J Bosseray A Brion JP Micoud M Besson G Chorea in a child with Churg-Strauss syndrome Stroke 1993 1 5 10.1161/01.str.24.8.1263 8342206

[B57] Rolla G Tartaglia N Motta M Warning nonrespiratory symptoms in asthma: catastrophic abdominal involvement in a case of Churg-Strauss syndrome Ann Allergy Asthma Immunol 2007 1 5 10.1016/S1081-1206(10)60743-0 17601277

[B58] Frayha RA Churg-Strauss syndrome in a child J Rheumatol 1982 1 5 6129323

[B59] Delplace D da Costa L Goffin L Oral ulceration: an unusual manifestation of Churg-Strauss syndrome J Eur Acad Dermatol Venereol 2007 1 5 10.1111/j.1468-3083.2007.02380.x 17659008

[B60] Sevinc A Hasanoglu HC Gokirmak M Yildirim Z Baysal T Mizrak B Allergic granulomatosis and angiitis in the absence of asthma and blood eosinophilia: a rare presentation of limited Churg-Strauss syndrome Rheumatol Int 2004 1 5 10.1007/s00296-003-0412-2 14628152

[B61] Sharma BK Daga MK Sharma M A limited form of Churg-Strauss syndrome presenting without asthma and eosinophilia Med J Aust 2004 1 5 10.5694/j.1326-5377.2004.tb06408.x 15516195

[B62] Masi AT Hunder GG Lie JT The American College of Rheumatology 1990 criteria for the classification of Churg-Strauss syndrome (allergic granulomatosis and angiitis) Arthritis Rheum 1990 1 5 10.1002/art.1780330806 2202307

[B63] Jennette JC Falk RJ Andrassy K Nomenclature of systemic vasculitides. Proposal of an international consensus conference Arthritis Rheum 1994 1 5 10.1002/art.1780370206 8129773

[B64] Ledford DK Vasculitis World Allergy Organization: Allergic Diseases Resource Center Available at: http://www.worldallergy.org/professional/allergic_diseases_center/vasculitis/. Accessed August 18, 2007

[B65] Xiao H Heeringa P Hu P Antineutrophil cytoplasmic autoantibodies specific for myeloperoxidase cause glomerulonephritis and vasculitis in mice J Clin Invest 2002 1 5 10.1172/JCI120039 PMC151154 12370273

[B66] Pfister H Ollert M Fröhlich LF Antineutrophil cytoplasmic autoantibodies against the murine homolog of proteinase 3 (Wegener autoantigen) are pathogenic in vivo Blood 2004 1 5 10.1182/blood-2004-04-1501 15150076

[B67] Schmitt WH Csernok E Kobayashi S Klinkenborg A Reinhold-Keller E Gross WL Churg-Strauss syndrome: serum markers of lymphocyte activation and endothelial damage Arthritis Rheum 1998 1 5 10.1002/1529-0131(199803)41:3<445::AID-ART10>3.0.CO;2-3 9506572

[B68] Chumbley LC Harrison EG Jr DeRemee RA Allergic granulomatosis and angiitis (Churg-Strauss syndrome). Report and analysis of 30 cases Mayo Clin Proc 1977 1 5 18640

[B69] Choi YH Im J-G Han BK Kim J-H Lee KY Myoung NH Thoracic manifestations of Churg-Strauss syndrome: radiologic and clinical findings Chest 2000 1 5 10.1378/chest.117.1.5 10631208

[B70] Worthy SA Müller NL Hansell DM Flower CD Churg-Strauss syndrome: the spectrum of pulmonary CT findings in 17 patients Am J Roentgenol 1998 1 5 10.2214/ajr.170.2.9456932 9456932

[B71] Moritani T Hiwatashi A Shrier DA Wang HZ Numaguchi Y Westesson PL CNS vasculitis and vasculopathy: efficacy and usefulness of diffusion-weighted echoplanar MR imaging Clin Imaging 2004 1 5 10.1016/S0899-7071(03)00191-8 15246475

[B72] Guillevin L Lhote F Gayraud M Prognostic factors in polyarteritis nodosa and Churg-Strauss syndrome. A prospective study in 342 patients Medicine (Baltimore) 1996 1 5 10.1097/00005792-199601000-00003 8569467

[B73] Hellmich B Gross W Recent progress in the pharmacotherapy of Churg-Strauss syndrome Expert Opin Pharmacother 2004 1 5 10.1517/14656566.5.1.5 14680433

[B74] Guillevin L Cevallos R Durand-Gasselin B Lhote F Jarrousse B Callard P Treatment of glomerulonephritis in microscopic polyangiitis and Churg-Strauss syndrome. Indications of plasma exchanges, meta-analysis of 2 randomized studies on 140 patients, 32 with glomerulonephritis Ann Med Interne (Paris) 1997 1 5 9255326

[B75] Jayne DR Chapel H Adu D Intravenous immunoglobulin for ANCA-associated systemic vasculitis with persistent disease activity QJM 2000 1 5 10.1093/qjmed/93.7.433 10874052

[B76] Orange JS Hossny EM Weiler CR Use of intravenous immunoglobulin in human disease: a review of evidence by members of the Primary Immunodeficiency Committee of the American Academy of Allergy, Asthma and Immunology J Allergy Clin Immunol 2006 1 4 suppl 5 10.1016/j.jaci.2006.01.015 16580469

[B77] Lamprecht P TNF-alpha inhibitors in systemic vasculitides and connective tissue diseases Autoimmun Rev 2005 1 5 10.1016/j.autrev.2004.06.001 15652776

[B78] Huugen D Cohen Tervaert JW Heeringa P TNF-alpha bioactivity- inhibiting therapy in ANCA-associated vasculitis: clinical and experimental considerations Clin J Am Soc Nephrol 2006 1 5 10.2215/CJN.02181205 17699331

[B79] Booth AD Jayne DR Kharbanda RK Infliximab improves endothelial dysfunction in systemic vasculitis: a model of vascular inflammation Circulation 2004 1 5 10.1161/01.CIR.0000111125.72039.83 15037536

[B80] Tiliakos A Shaia S Hostoffer R Kent L The use of infliximab in a patient with steroid-dependent Churg-Strauss syndrome J Clin Rheumatol 2004 1 5 10.1097/01.rhu.0000130684.35729.55 17043477

[B81] Kaushik VV Reddy HV Bucknall RC Successful use of rituximab in a patient with recalcitrant Churg-Strauss syndrome Ann Rheum Dis 2006 1 5 10.1136/ard.2005.047308 PMC1798243 16837496

[B82] Tatsis E Schnabel A Gross WL Interferon-alpha treatment of four patients with the Churg-Strauss syndrome Ann Intern Med 1998 1 5 10.7326/0003-4819-129-5-199809010-00004 9735064

[B83] Assaf C Mewis G Orfanos CE Geilen CC Churg-Strauss syndrome: successful treatment with mycophenolate mofetil Br J Dermatol 2004 1 5 10.1111/j.1365-2133.2003.05807.x 15030353

[B84] Giavina-Bianchi P Giavina-Bianchi M Agondi R Kalil J Administration of anti-IgE to a Churg-Strauss syndrome patient Int Arch Allergy Immunol 2007 1 5 10.1159/000103228 17536214

[B85] Booth AD Almond MK Burns A Pan-Thames Renal Research Group. Outcome of ANCA-associated renal vasculitis: a 5-year retrospective study Am J Kidney Dis 2003 1 5 10.1016/s0272-6386(03)00025-8 12666064

[B86] Van Dellen RG Can you top this? 37-year survival of patient with Churg-Strauss syndrome Ann Allergy Asthma Immunol 2004 1 5 10.1016/S1081-1206(10)61703-6 15237772

